# miR-34a exerts as a key regulator in the dedifferentiation of osteosarcoma via PAI-1–Sox2 axis

**DOI:** 10.1038/s41419-018-0778-4

**Published:** 2018-07-10

**Authors:** Yu Zhang, Yubin Pan, Chunyuan Xie, Yan Zhang

**Affiliations:** Key Laboratory of Gene Engineering of the Ministry of Education, State Key Laboratory of Biocontrol, School of Life Sciences, Sun Yat-sen University, Guangzhou, 510006 China

## Abstract

Osteosarcoma (OS) is a malignant bone cancer with severe chromosomal abnormalities and genetic aberrations. Our previous work reported the dedifferentiation of OS, which is related to poor prognosis. However, the molecular mechanism that regulates OS dedifferentiation is still a subject of exploration. Emerging evidence has suggested that microRNAs (miRNAs) are associated with the pathogenesis of OS and could potentially be developed for use as diagnostic biomarkers and therapeutic strategies. In the present study, we intended to illustrate the role of miR-34a in the dedifferentiation of OS. Upregulation of miR-34a was observed while OS cells were induced into stem-like phenotype. Notably, inhibition of miR-34a could promote the reprogramming transition of OS. Further exploration on the downstream network of miR-34a identified that blocking plasminogen activator inhibitor-1 (PAI-1) expression could restrain OS dedifferentiation into cancer stem-like cells by downregulating SRY-related-HMG box (Sox) 2. We also showed that Sox2 overexpression rescued the suppression phenotype driven by PAI-1 inhibition. Conversely, PAI-1 inhibitor (PAI-039) could suppress the upregulation of Sox2 expression caused by miR-34a inhibition. Be applying bone extracellular matrix (BEM)-OS models, we demonstrated the phenotypic heterogeneity of OS cells, consistent with a strong concordance between PAI-1 and Sox2 expression levels. Taken together, our findings proved miR-34a to be a *bona fide* suppressor involved in the regulation of OS dedifferentiation. Targeting miR-34a or its direct target PAI-1 could offer new strategies for OS treatment.

## Introduction

Osteosarcoma (OS) is the most common malignant primary bone tumor in childhood and adolescents, and represents the second highest cause of cancer-related death in children and young adults. It features rapid development, local recurrence, strong metastatic ability and poor prognosis^1^.

Primary chemotherapy, tumor excision and adjuvant chemotherapy are the most commonly used treatments for OS^2^. Despite advanced multiagent neoadjuvant and adjuvant chemotherapies, the clinical outcome for patients with OS remains discouraging, and the long-term survival rate for high-grade OS is poor. The genomic complexity and the inter-/intra- tumoral heterogeneity of OS has severely hampered the efficiency of molecular therapeutic targets^3^. There is an urgent need to identify innovative treatment strategy.

The microenvironment of OS is dynamic and variable, with complex bone extracellular matrix (ECM) and diverse populations of localized cells. Our previous study has revealed that abundant transforming growth factor β1 (TGFβ1) and hypoxic environment could induce OS cell toward a cancer stem cell (CSC) phenotype, which was termed as sarcosphere. Gene set enrichment analysis (GSEA) revealed that gene alterations during the process of dedifferentiation were closely correlated with chemoresistance and metastasis in OS patients^4^.

OS results from multiple factors and gene aberrations^5^. Up to 22% of OS patients carry an abnormal *TP53* gene, and the allelic loss on chromosome 17p13 is confirmed in 75% of patients by a detection of the mutation in the germline^6,7^. As a direct transcriptional target of p53, microRNA-34a (miR-34a) was found to be decreased and undergo minimal deletions and epigenetic inactivation in OS cells^8^. Several studies have demonstrated that miR-34a is involved in chemoresistance, proliferation, and metastasis of OS^9–11^. Importantly, miR-34 family members, including miR-34a, miR-34b and miR-34c have been proven to be the key regulators that suppress reprogramming downstream of p53. Among all these members, miR-34a presented the highest p53-dependent induction level during reprogramming. The deficiency of miR-34a altered mouse embryonic fibroblast (MEF) reprogramming by posttranscriptional derepression of pluripotency genes^12^. miR-34a also regulated the asymmetric division of colon CSCs^13^ and inhibited breast cancer stemness^14^. These finding provided strong evidence that manipulating specific microRNA (miRNA) functions could be a promising strategy in modulating pluripotency.

In the present study, we aimed to elucidate the molecular mechanism in the regulation of OS dedifferentiation with a focus on miR-34a. Results showed that the expression of miR-34a, which developed its specific tendency in different phases of OS differentiation, might inhibit the dedifferentiation of OS. We identified a novel candidate among potential targets of miR-34a, plasminogen activator inhibitor-1 (PAI-1, also termed as Serpin Family E Member 1, SERPINE1). Inhibiting PAI-1 expression could suppress OS dedifferentiation by downregulating SRY-related-HMG box (Sox) 2. We herein demonstrated that p53-regulated miR-34a served as a crucial regulator in OS by inhibiting dedifferentiation via the downregulation of PAI-1-Sox2 axis.

## Materials and Methods

### Cell lines

MNNG/HOS, MG-63 and U-2 OS human OS cell lines were obtained from cell bank of the Chinese Academy of Sciences (Shanghai, China, http://www.cellbank.org.cn). All the cell lines were confirmed to be free from bacteria and mycoplasma contamination and authenticated by cellular morphology and short tandem repeat analysis. All the cells were incubated at 37 °C in a humidified atmosphere containing 5% CO_2_. MNNG/HOS and MG-63 were maintained in Dulbecco’s Modified Eagle’s Medium/F12 (DF12) containing 5% FBS. U-2 OS was maintained in DF12 containing 10% FBS. The sarcospheres were cultured in serum-free DF12 supplemented with 5 factors (5 F), including 10 μg/ml human insulin, 5 μg/ml human transferrin, 10 μM 2-aminoethanol, 10 nM sodium selenite, and 10 μM mercaptoethanol as described previously^4^.

### Vectors, oligonucleotides and cell transfection

The silencing of miR-34a gene was performed using lentiCRISPRv1 vector (Addgene Plasmid 49535) with a puromycin selectable markers. The gRNA sequences are as follow: 5′-CACCGGTTGTTGTGAGCAATAGTA-3′ and 5′-AAACTACTATTGCTCACAACAACC-3′.

Synthetic mimic, inhibitor, small interfering RNAs (siRNAs) and the corresponding negative control (NC) oligonucleotides were purchased from RiBoBio (Guangzhou, China). The siRNAs targeting the mRNA of human PAI-1 (GenBank accession no. NM000602) were indicated as siPAI-1. The NC for the miR-34a-5p mimic, miR-34a-5p inhibitor and the siRNAs were non-homologous to any human genome sequences. All oligonucleotides are listed in Supplementary Table S1. A concentration of 50 nM miR-34a-5p mimic, 100 nM miR-34a-5p inhibitor, 200 nM siPAI-1 and the corresponding NC were transfected with Lipofectamine 3000 (Life Technologies) according to the manual. The transfection and silencing efficiency were determined by real time reverse transcriptase polymerase chain reaction (qRT-PCR).

For dual-luciferase reporter assay, the plasmid of pGL3-PAI-1 was generated by inserting the 3’UTR of PAI-1 into pGL3-control vector digested with XbaI and Eco32I (EcoRV).

The stable overexpression of Sox2 in OS cells was achieved by using pMXs retroviral expression vector (Addgene plasmid 17218).

### qRT-PCR

Total RNA was extracted by Trizol reagent (Magen) and reverse-transcribed using First Strand cDNA Synthesis Kit ReverTra Ace -α- (TOYOBO). PCR amplification was performed with LightCycler® 480 SYBR Green I Master (Roche). The expression levels were normalized to U6 (for miRNA) or GAPDH (for mRNA). Primer sequences for qRT-PCR are available in Supplementary Table S2.

### Luciferase reporter assay

OS cells were transfected with 100 nM miR-34a-5p mimic or mimic NC for 24 h and subsequently co-transfected with *Renilla* luciferase control vector and wild-type (WT) or mutant (MUT) pGL3-PAI-1. Luciferase assay were performed 48 h post transfection, using Dual-Luciferase® Reporter Assay System (Promega). Firefly luciferase activity was normalized to *Renilla* luciferase activity.

### Western blot analysis

Cells were resuspended in lysis buffer and incubated on ice for 30 min. The cell suspension was homogenized and then centrifuged at 12,000 r.p.m. for 10 min at 4 °C. The supernatant was transferred to a new tube and denatured at 95 °C for 10 min before loading. Equal amounts of protein were separated in 10% SDS-polyacrylamide gel and then transferred to PVDF membranes (GE Healthcare). The primary antibodies used in this study included: anti-GAPDH antibody and anti-Sox2 antibody from Cell Signaling Technology, anti-PAI-1 antibody from Santa Cruz. Both anti-mouse and anti-rabbit secondary antibodies were from Abcam.

### Colony forming assay

Single WT U-2 OS or miR-34a-depleted U-2 OS cells were plated onto 0.66% solidified agar-based six-well plates, respectively. Soft agar cultures were incubated for 14 days and fed with 1 mL medium at day 7. The forming colonies were fixed with 4% paraformaldehyde (PFA) for 20 min at room temperature and stained with crystal violet.

### Adipogenesis differentiation and Oil Red O staining

WT U-2 OS or miR-34a-depleted U-2 OS cells were plated onto 24-well culture plates and were kept in adipogenic medium using StemPro® Adipogenesis Differentiation Kit (Life Technologies). After cultured for 12 days, media were removed from the culture plates and cells were rinsed once with phosphate buffered saline (PBS), fixed with 4% PFA for 20 min. The cells were then rinsed twice and analyzed with Oil Red O staining kit (BASO). Hematoxylin was applied as a nuclear counterstain.

### Three-dimensional (3D) culture using bone extracellular matrix (BEM) scaffolds

Mouse bone was aseptically excised from the hindlimb and rinsed with PBS twice. After decalcification and decellularization with reference to current approaches^15,16^, the OS cells were then injected from proximal or distal epiphysis when the needle of sterile insulin syringe reached the medullary cavity of BEM. It would take 4 h for OS cells to attach to the BEM, and then the medium was added into the plate. After cultured for 14 days, BEM-OS was fixed in 10% buffered formalin for histological identification.

### Hematoxylin-eosin (H&E) staining and immunohistochemistry (IHC)

The tissues were fixed in 10% buffered formalin for 24 h, embedded in paraffin, and then sliced into 3–5 μm sections. After deparaffinization, sections were stained with H&E or were processed for IHC using anti-PAI-1 antibody (Santa Cruz) or anti-Sox2 antibody (Cell Signaling Technology). After staining, sections were dehydrated, cleared with xylene and mounted with resinene.

### Statistical analysis

All the experiments were carried out at least three times independently. The data were presented as the mean ± SEM (SD) and the statistical analyses were performed using either Student’s *t* test or one-way ANOVA. A value of *P* < 0.05 was considered to be statistically significant (*P < 0.05, **P < 0.01, ***P < 0.001, #*P* > 0.05).

## Results

### miR-34a was upregulated during the dedifferentiation of OS

Our previous study demonstrated that OS cells cultured in serum-free medium supplemented with TGFβ1 could undergo a gradual morphological transition from adherent cells to spheroid cells (Supplementary Fig. S1a, b), along with the activation of Sox2 (Supplementary Fig. S1c-e). Besides, the expression of NANOG and OCT4 was also induced during the formation of stem-like spheroid cells. These spheroid cells expressed human embryonic stem cell-specific surface antigens, including stage-specific embryonic antigen 1 (SSEA-1), stage-specific embryonic antigen 4 (SSEA-4), tumor rejection antigen 1-60 (TRA-1-60), and tumor rejection antigen 1-81 (TRA-1-81) at much higher levels than adherent cells^4^. To prove dedifferentiation more sufficiently, we analyzed our microarray data (GSE38135), which was performed to evaluate gene alterations that might be closely related with OS dedifferentiation. During the process of dedifferentiation, multiple genes that were demonstrated to be pivotal in cancer stem cell properties were induced, including PDGFRB, NOTCH1, JAG1, HES1, and HEY1 of the Notch signaling, SNAI1 and SNAI2^17–20^. qRT-PCR confirmed the results of microarray analysis (Supplementary Fig. S1f, g). What draws our attention is that different OS cell line exhibits different transition capacity. Among the three OS cell lines, MNNG/HOS could form sarcospheres with a higher efficacy than MG-63, whereas U-2 OS was not able to dedifferentiate (data not shown).

OS is a malignant tumor with high frequency of p53 abnormality and presents heterogeneous p53 phenotypes^6,7^. Strong evidence has suggested that p53 regulates the genomic stability, proliferation and immune properties of bone mesenchymal stem cells (MSCs). p53 loss-of-function in MSCs compromises osteogenic differentiation and significantly influences bone tumor microenvironment, both of which have a marked impact on the development of OS^21^. For confirming p53 functional status in OS cells, we treated “p53-mutant” MNNG/HOS, “p53-null” MG-63 and “p53-wild type” U-2 OS with 10 μM Nutlin-3 for 48 h and observed the response of these three kinds of OS cells^22,23^. As a potent inhibitor of p53-Mdm2 interaction, Nutlin-3 can stabilize p53, leading to cell cycle arrest, apoptosis, and growth inhibition of human tumor xenografts^24^. As expected, only “p53-wild type” U-2 OS was induced into a growth-inhibiting state after Nutlin-3 treatment (Supplementary Fig. S2a, b).

**Fig. 1:**
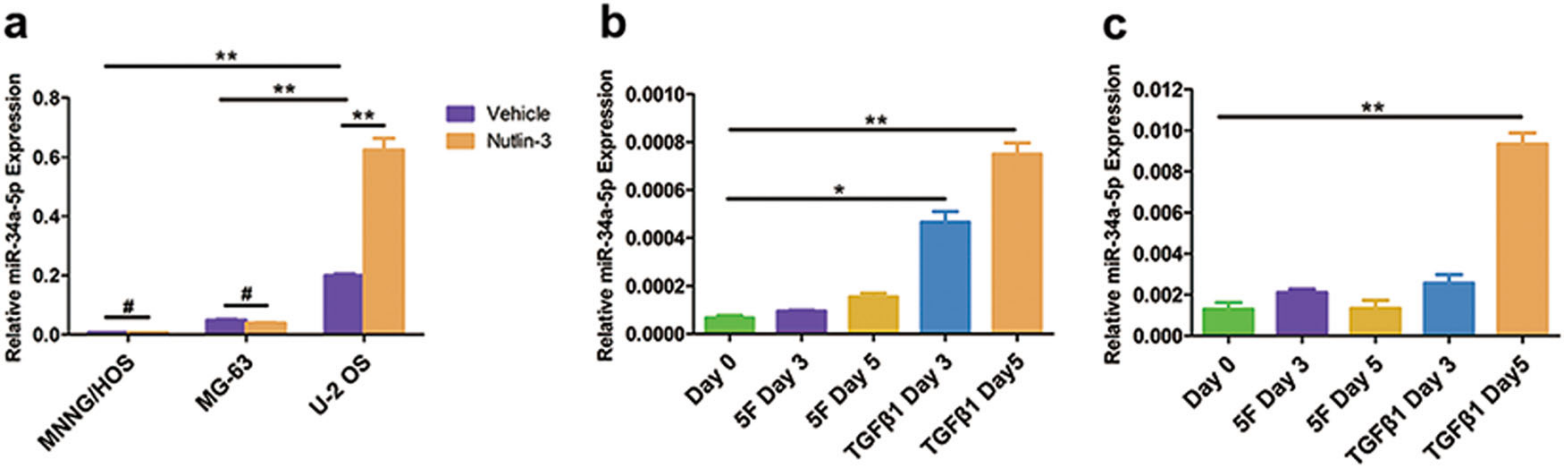
miR-34a was correlated with the dedifferentiation of OS cells. **a** miR-34a-5p expression was increased by 10 μM Nutlin-3 treatment in a p53-dependent manner. **b**, **c** miR-34a-5p was upregulated during the dedifferentiation of MNNG/HOS (**b**) and MG-63 (**c**). *P < 0.05, **P < 0.01, #P > 0.05

Downstream factors of p53 are known to play essential roles in regulating the reprogramming efficiency^12,25^, of which miR-34 family gets our attention. The pri-miRNA expression of miR-34a was dramatically induced during OS dedifferentiation. Other members of miR-34 family, pri-miR-34b/c, presented much lower expression level than pri-miR-34a and show no significantly change during this process (Supplementary Fig. S2c–f). We then analyzed the expression of mature miR-34a in three OS cell lines after Nutlin-3 treatment. Upregulation of miR-34a was only detected in U-2 OS, suggesting that miR-34a expression was increased by Nutlin-3 treatment in a p53-dependent manner (Fig. 1a). Interestingly, among the three OS cell lines, “p53-wild type” U-2 OS displayed the highest level of miR-34a, while “p53-mutant” MNNG/HOS was the lowest. Distinct levels of miR-34a seemed to be correlated to the ability of dedifferentiation.

To determine the role of miR-34a in OS dedifferentiation, we examined miR-34a expression levels and found that miR-34a was upregulated during the dedifferentiation of MNNG/HOS (Fig. 1b) and MG-63 (Fig. 1c). TGFβ1, as an important microenvironmental signal of OS, dramatically promoted the expression of miR-34a. We assumed that miR-34a might serve as a driver or a negative feedback controller in the dedifferentiation of OS.

### miR-34a suppressed the dedifferentiation of OS

**Fig. 2:**
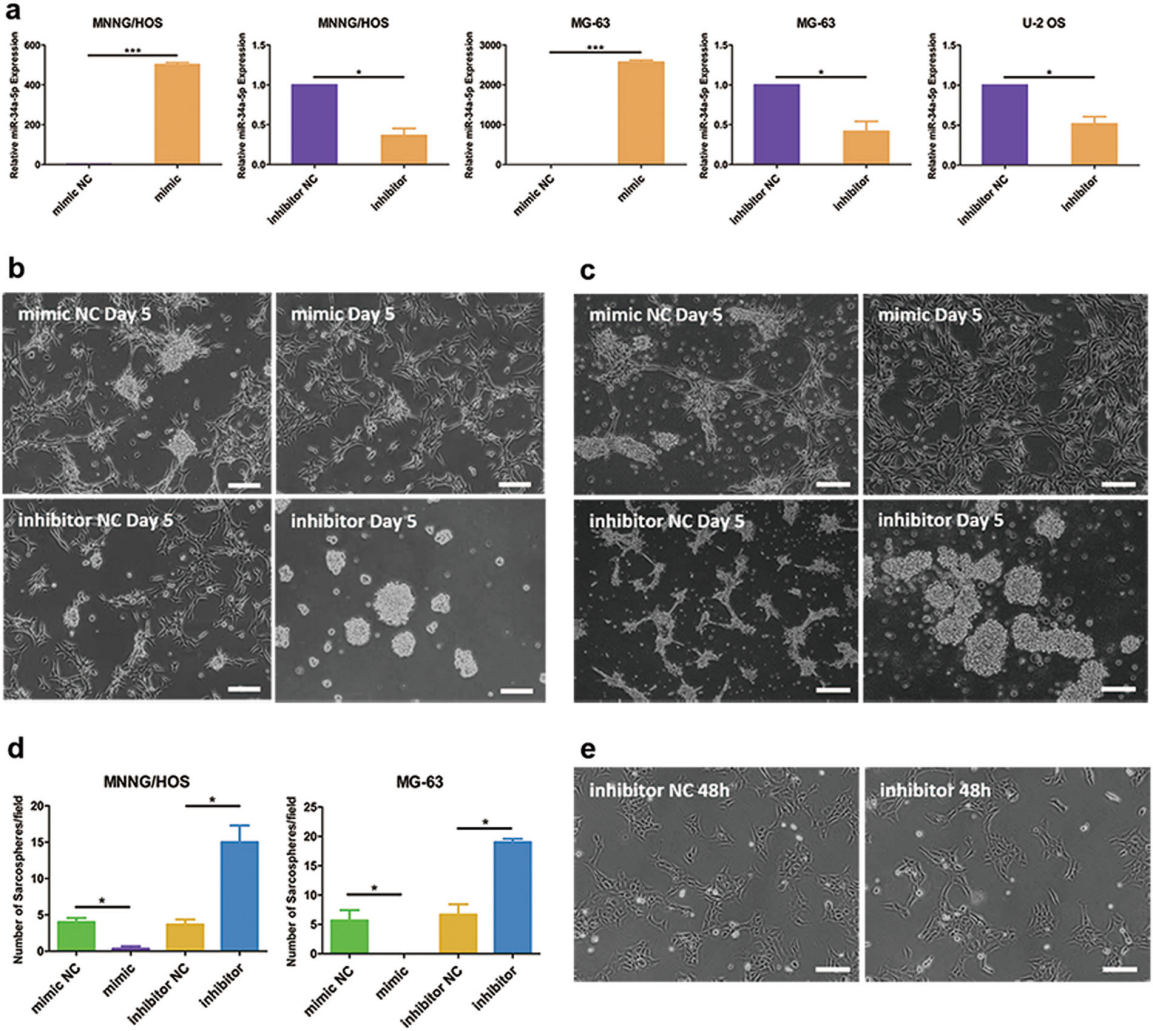
miR-34a could repress the dedifferentiation of MNNG/HOS and MG-63 cells. **a** Overexpression and inhibition of miR-34a-5p in OS cells. **b**, **c** Representative images of the capacity of spherogenicity of MNNG/HOS (**b**) and MG-63 (**c**) after transfected with miR-34a-5p mimic, miR-34a-5p inhibitor and the corresponding NC. **d** Statistical analysis of the number of sarcospheres during dedifferentiation process. The number of sarcospheres was counted under ×40 microscope. **e** Morphological changes of U-2 OS cells in serum-free medium supplemented with TGFβ1. Scale bar = 100 μm. *P < 0.05, ***P < 0.001

To confirm the exact role in the dedifferentiation of OS, we manipulated the expression of miR-34a by using chemically synthesized oligonucleotides. miR-34a-5p mimic, miR-34a-5p inhibitor and the corresponding NC were transiently transfected into OS cells (Fig. 2a). miR-34a could repress the dedifferentiation in MNNG/HOS (Fig. 2b) and MG-63 (Fig. 2c). Inhibition of miR-34a accelerated the forming of sarcospheres, and dramatically increased both the number and size of sarcospheres (Fig. 2d). Yet, the inhibition of miR-34a in U-2 OS could not promote the forming of spheroid cells (Fig. 2e). We speculated that miR-34a could provide a barrier for cancer cell reprogramming.

### Knockout of miR-34a promoted the dedifferentiation of OS

**Fig. 3:**
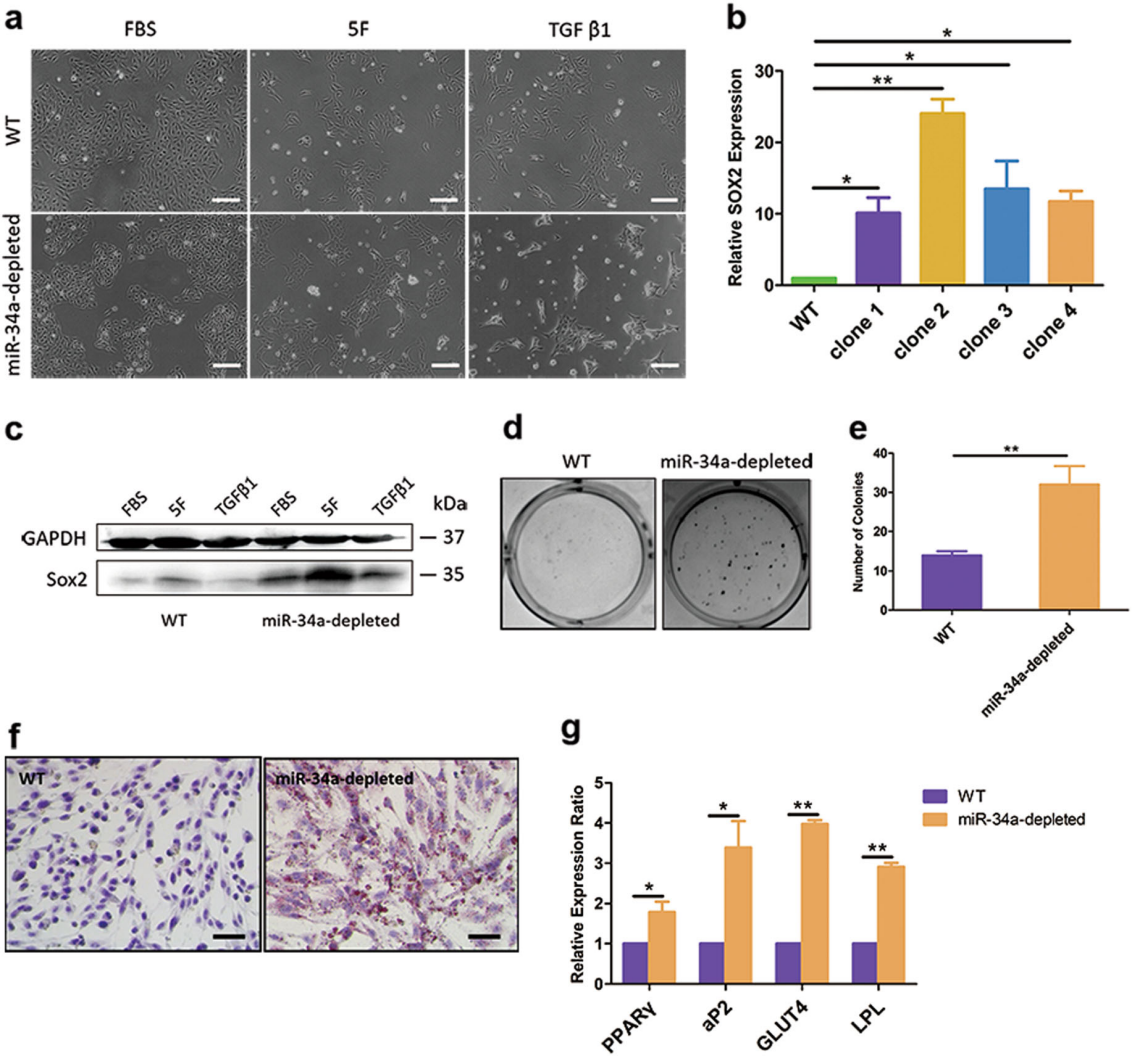
Knockout of miR-34a could promote the dedifferentiation of U-2 OS cells. **a** Morphological changes in miR-34a-depleted U-2 OS compared to WT U-2 OS. **b**, **c** qRT-PCR (**b**) and western blotting (**c**) analysis of Sox2 expression in WT U-2 OS and miR-34a-depleted U-2 OS. **d**, **e** Soft agar colony formation assay to investigate the self-renewal ability of WT U-2 OS cells and miR-34a-depleted U-2 OS. **f** Adipogenic differentiation of WT U-2 OS cells and miR-34a-depleted U-2 OS. **g** qRT-PCR analysis of adipogenic markers PPARγ, aP2, GLUT4 and LPL mRNA expression level in WT U-2 OS cells and miR-34a-depleted U-2 OS after adipogenic differentiation. Scale bar = 100 μm. *P < 0.05, **P < 0.01

For a more stable and efficient inhibitory strategy, lenti-CRISPR system was employed for miR-34a depletion. We obtained positive clones via puromycin selection, which were further confirmed by T7EI assay and sequencing (Supplementary Fig. S3a, b). Unlike WT U-2 OS, miR-34a-depleted U-2 OS presented polygonal and spindle-like cell clusters under serum-free medium with TGFβ1 (Fig. 3a). Detection on Sox2 expression (Fig. 3b, c) and colony formation capacity (Fig. 3d, e) suggested that miR-34a-depleted U-2 OS harbored a stronger self-renewal ability than WT U-2 OS. Furthermore, we detected the differentiation capacity into adipocytes of U-2 OS cells after adipogenic differentiation. miR-34a depleted U-2 OS cells had morphological changes from elongated mesenchymal-like shape into oval shape. Oil Red O staining analysis revealed that mature adipocytes were differentiated from miR-34a-depleted U-2 OS cells, while WT U-2 OS could not form lipid vacuoles in adipogenic differentiation medium (Fig. 3f). Adipocyte differentiation was also proved by detecting the expression of adipogenic transcription factors and lipid accumulation genes. The expression of the adipogenic markers peroxisome proliferator-activated receptor γ (PPARγ), fatty acid-binding protein 4 (FABP4, also known as aP2), lipoprotein lipase (LPL) and glucose transporter 4 (GLUT4) were also upregulated in miR-34a-depleted U-2 OS in comparison with WT U-2 OS after adipogenic differentiation (Fig. 3g). Therefore, it could be assumed that miR-34a depletion enhanced the capability of reprogramming in OS cells.

### PAI-1 was directly targeted by miR-34a and functionally involved in the regulation of OS dedifferentiation

**Fig. 4:**
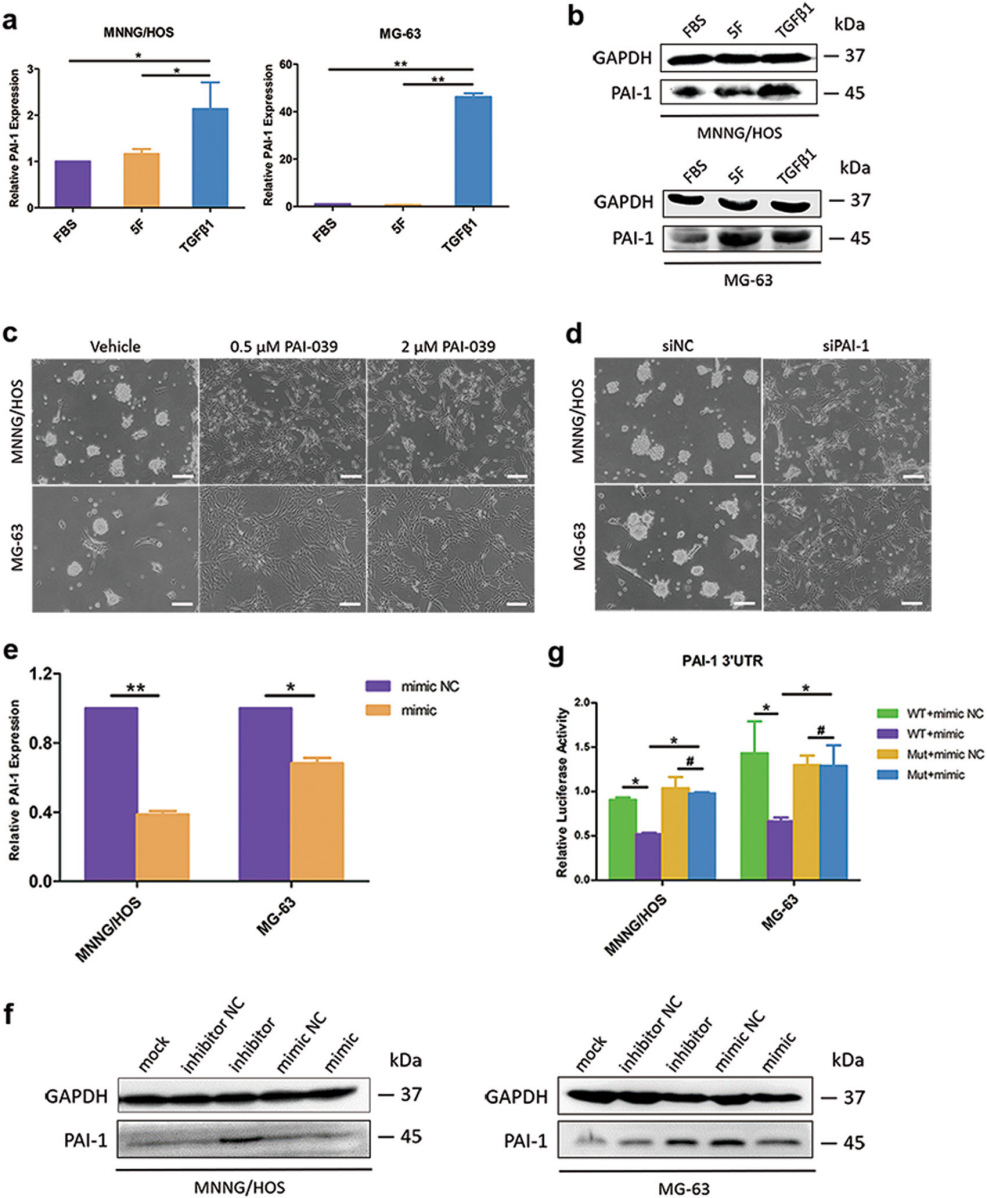
PAI-1 was a target of miR-34a and could be involved in the regulation of OS dedifferentiation. **a**, **b** Both mRNA (**a**) and protein (**b**) expression levels of PAI-1 were upregulated during the dedifferentiation of MNNG/HOS and MG-63 cells. **c**, **d** Inhibition of PAI-1 by PAI-039 treatment (**c**) or siRNA transfection (**d**) could suppress the forming of sarcospheres. Scale bar = 100 μm. **e** qPCR analysis of PAI-1 mRNA expression in MNNG/HOS and MG-63 cells after transfected with miR-34a-5p mimic or mimic NC. **f** Immunoblotting of PAI-1 in five groups of MNNG/HOS and MG-63 cells (mock, inhibitor NC, miR-34a-5p inhibitor, mimic NC and miR-34a-5p mimic). **g** Overexpression of miR-34a-5p significantly suppressed the luciferase activity of the PAI-1 WT 3′-UTR luciferase reporter construct but had no effect on the mutant construct. *P < 0.05, **P < 0.01, #*P* > 0.05

Our previous microarray analysis (GSE38135) showed that multiple genes were significantly changed during the dedifferentiation of OS^4^. PAI-1 emerged as an eye-catching role worth to explore its regulation of OS dedifferentiation (Supplementary Fig. S4a). The expression level of PAI-1 was significantly upregulated during OS dedifferentiation (Fig. 4a, b). To understand the role of PAI-1 in OS dedifferentiation, we suppressed the expression of PAI-1 by inhibitor PAI-039 (Tiplaxtinin) treatment or siRNA transfection (Supplementary Fig. S4b). Inhibition of PAI-1 suppressed the forming of sarcospheres (Fig. 4c, d).

On the basis of both Targetscan and miRbase database prediction, we found that miR-34a seed region matches in the 3’UTR of PAI-1 (Supplementary Fig. S4c). Levels of PAI-1 were downregulated by miR-34a overexpression and were upregulated by miR-34a inhibition (Fig. 4e, f). PAI-1-3′-UTR luciferase activities were also suppressed in response to miR-34a overexpression (Fig. 4g). The suppressive effect on PAI-1 3′-UTR was rescued by several nucleotide substitutions in the binding sites as shown in Supplementary Fig. S4c. These results further corroborated that PAI-1, targeted by miR-34a, might be closely associated with the transition from adherent cells to spheroid cells.

### Inhibition of PAI-1 suppressed OS dedifferentiation via Sox2 downregulation

**Fig. 5:**
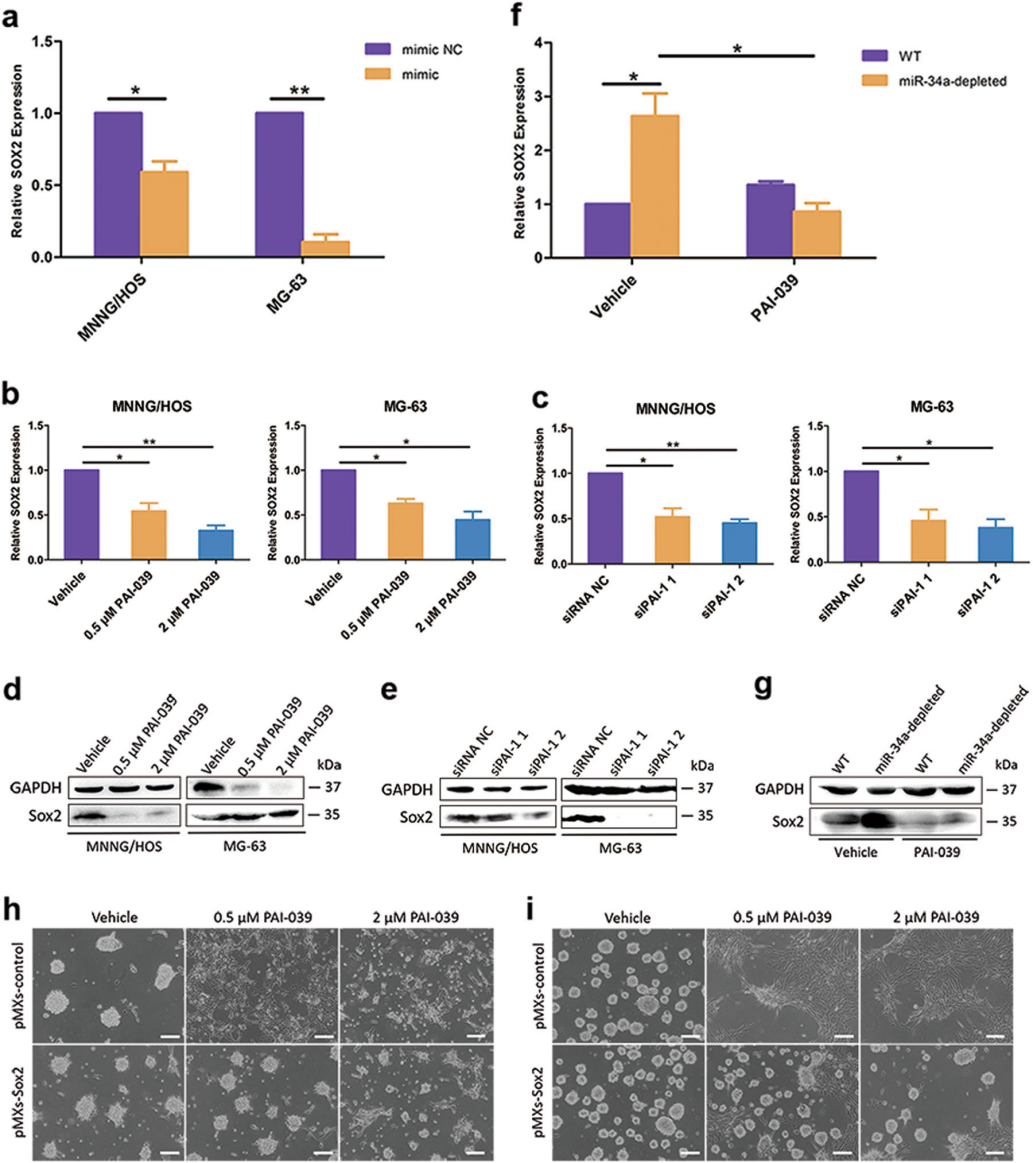
Inhibition of PAI-1 suppressed OS dedifferentiation via Sox2 downregulation. **a** The mRNA levels of Sox2 were downregulated after miR-34a overexpression in OS cells. **b**–**e** The inhibition of PAI-1 by PAI-039 (**b**, **d**) or siRNAs (**c**, **e**)-mediated gene silencing could downregulate the mRNA (**b**, **c**) and protein (**d**, **e**) levels of Sox2 in both MNNG/HOS and MG-63 cells. **f**, **g** PAI-039 could reduce both mRNA (**f**) and protein (**g**) expression level of Sox2 promoted by miR-34a downregulation. **h**, **i** Representative images showing that Sox2 overexpression could rescue the suppression of MNNG/HOS (**h**) and MG-63 (**i**) dedifferentiation caused by PAI-1 inhibition. Scale bar = 100 μm. *P < 0.05, **P < 0.01

It has been demonstrated that PAI-1 inhibitor could decrease the activity of the core promoter and enhancer of Sox2 gene in tumor-initiating stem cells (TICs) of head and neck cancer^26^. We found that the expression of stemness gene Sox2 was notably elevated during the dedifferentiation of MNNG/HOS and MG-63 (Supplementary Fig. S1c-e). Also, the mRNA level of SOX2 was mediated by miR-34a expression, in accordance with the response of PAI-1 (Fig. 5a). Manipulating the expression of PAI-1 by PAI-039 treatment or siRNA transfection both downregulated Sox2 expression (Fig. 5b–e). Additionally, PAI-039 treatment could reduce Sox2 expression promoted by miR-34a depletion (Fig. 5f, g). To determine that the effects of PAI-1 on the dedifferentiation were mediated by Sox2, we employed a retroviral construct to stably overexpress Sox2 in MNNG/HOS and MG-63 cells (Supplementary Fig. S5a, b). Results indicated that Sox2 overexpression could rescue the suppression of dedifferentiation caused by PAI-1 inhibition (Fig. 5h, i). Taken together, these results provided convincing support for the crucial role of Sox2 mediated by miR-34a–PAI-1 axis in the regulation of OS dedifferentiation.

### miR-34a correlated to OS phenotypic heterogeneity and PAI-1 expression was in conjunction with Sox2-positive cells

Current exploration of the molecular interactions during OS dedifferentiation implicated that bone microenvironment signals have made noticeable contributions. However, traditional two-dimensional (2D) culture on dishes could rarely recapitulate the intricate bone supporting construction in vivo. To reveal the dedifferentiation process in depth, we have generated tissue-derived BEM and established a 3D OS model in vitro that could mimic an intact OS environment. BEM maintained the structural characteristics of native mouse tibia and displayed amazing biocompatibility, providing a promising scaffold for the proliferation of injected OS cells (Supplementary Fig. S6a–d).

**Fig. 6:**
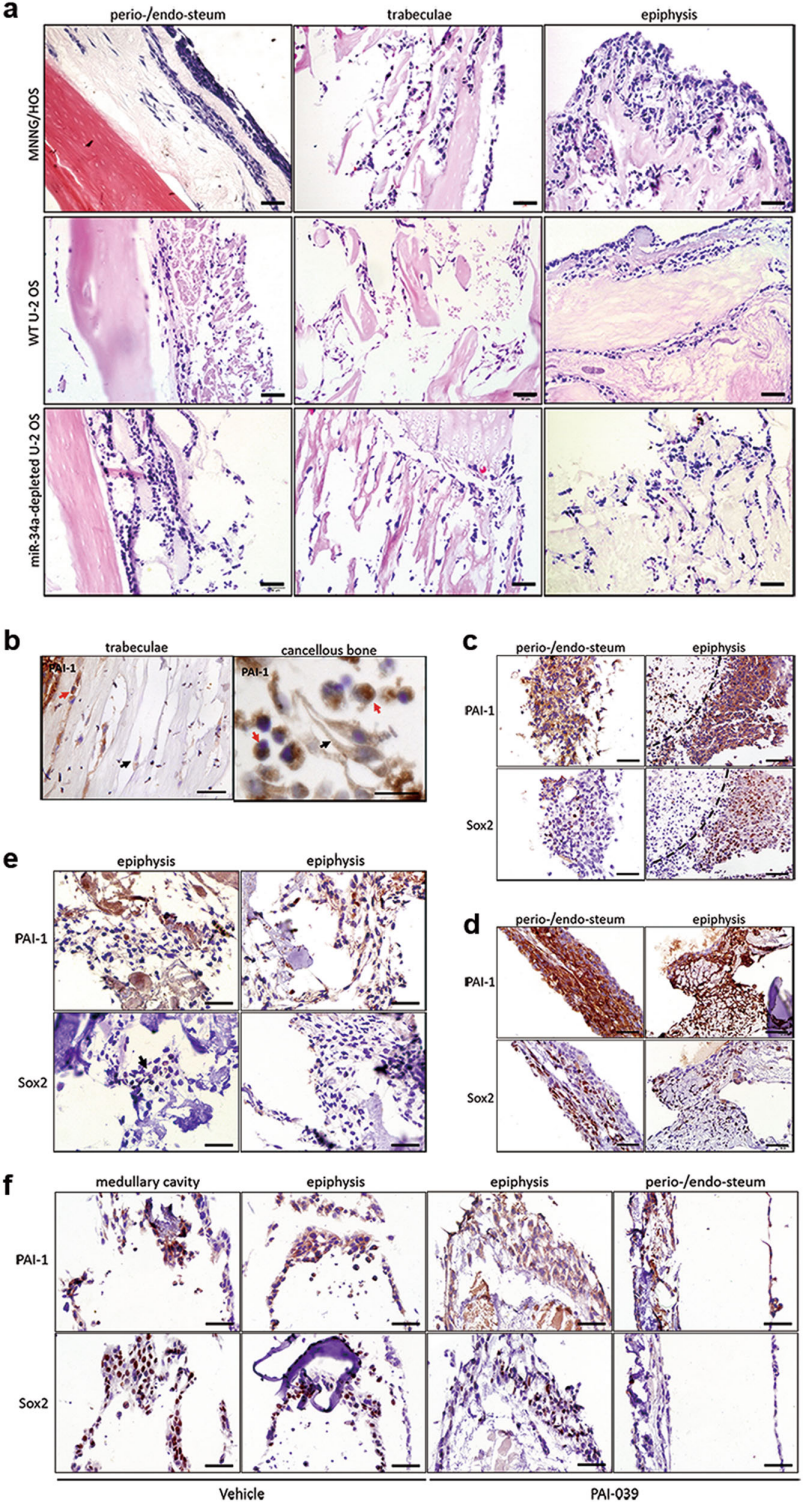
miR-34a expression correlated to OS phenotypic heterogeneity and high level of PAI-1 expression was in conjunction with Sox2-positive cells. (**a**) H&E analysis of the injected OS cells in BEM. Magnified images were shown as an inset. (**b**) IHC analysis of PAI-1 expression of injected MNNG/HOS cells in BEM. Black arrows pointed out the elongated MNNG/HOS cells and the red arrows pointed out the rounded ones. (**c, d**) IHC analysis of PAI-1 and Sox2 expression of MNNG/HOS adherent cells (**c**) and spheroid cells (**d**). The dotted line indicated the necrotic area and the asterisk denoted non-specific staining, a frequent problem in the apoptotic cells. (**e, f**) IHC analysis of PAI-1 and Sox2 expression of WT U-2 OS (**e**) and miR-34a-depleted U-2 OS treated with vehicle or PAI-039 (**f**). Black arrows indicated the site of Sox2-positive cell subpopulation. Magnified images were shown as an inset. Scale bar = 50 μm

Results showed that the BEM-OS model provided a proper explanation for phenotypic and functional heterogeneity among the OS cells (Fig. 6a). Unlike 2D culture, MNNG/HOS cells presented two typical morphologies, elongated mesenchymal-like shape and rounded shape, which was similar to the highly heterogeneous clinicopathological feature of OS tissues. The vast majority of elongated cells located in the bone periosteum with plenty of nutrients and oxygen, while the rounded cells rested in cancellous bone and medullary cavity with poor medium permeation. Strikingly, miR-34a-depleted U-2 OS performed significantly higher heterogeneity and stronger invasive ability, while WT U-2 OS basically remained its monolayer morphology and mostly colonized along BEM.

Apart from the morphological heterogeneity, the expression of PAI-1 and Sox2 in the BEM-OS were varied. In the trabecula or cancellous bone region, the expression of PAI-1 exhibited higher levels in rounded MNNG/HOS cells than the elongated ones (Fig. 6b). Sox2 expression was detected in both MNNG/HOS adherent cells (Fig. 6c) and spheroid cells (Fig. 6d) that were injected and cultured in BEM, which co-located to regions with elevated PAI-1 expression. Compared with MNNG/HOS adherent cells, MNNG/HOS spheroid cells presented higher PAI-1 expression and more Sox2-positive staining. The necrotic area, which was surrounded by PAI-1^+^/Sox2^+^ cells, could be discovered incidentally in the histological section of BEM (Fig. 6c).

In the BEM-OS model of U-2 OS, only a few WT U-2 OS cells in the bone epiphyseal plate region expressed Sox2 (Fig. 6e), while miR-34a-depleted U-2 OS displayed a significantly increasing population with immunostaining of Sox2, most of which were rounded cells in the medullary cavity. PAI-039 treatment could decrease the Sox2-positive subpopulation, which was only detected in the epiphysis site (Fig. 6f). Hence, the expression of Sox2 was accompanied by a concomitant expression of PAI-1 in BEM-OS models.

## Discussion

Stem cells existing in human adult tissues are responsible for homeostasis and restoration. Somatic cells could be artificially converted into induced pluripotent stem cells (iPSCs) by ectopic expression of certain pluripotency-associated factors and global epigenetic reprogramming^27,28^. Cancer cells can also acquire stem-like properties by several dedifferentiation inducers and microenvironmental signals^29^. The acquisition and accumulation of genetic and epigenetic alterations could fuel cell reprogramming and in turn imparts a permissive niche for tumor heterogeneity and further progression. It was suggested that Notch activity identified lung cancer cells with sphere formation and self-renewal ability. The Notch active cells had the capacity to form xenograft in the mouse model and were more resistant to chemotherapy exposure^17^. Some studies have also illustrated the inevitable role of extrinsic cues in the regulation of cancer stemness on many fronts. In colorectal cancer, tumor-associated myofibroblasts secreted factors such as HGF that stimulated Wnt signaling, which subsequently gave rise to cancer stem-like cells and tumorigenesis^30^.

Particularly, our previous research declared TGFβ1 and hypoxia as crucial microenvironmental factors that designated OS cells with promoted tumorigenicity, neo-vasculogenesis and metastatic potential^4^. Single-cell suspensions of OS could form spheroid cells in anchorage-independent and serum-free conditions. Compared with adherent cells, these spheroid cells showed higher expression of the pluripotency-related genes OCT4, NANOG and SOX2^4,31^. The dedifferentiation of OS was proved to account for tumor growth, metastasis and drug resistance^32,33^. Targeting the dedifferentiation process could yield new approaches to solve the ticklish problem such as unfavorable prognosis and relapse after therapy. Our previous research has emphasized the importance of microenvironmental signals and provided an exciting starting point to explore OS dedifferentiation in a clearer perspective. It would be of interest to further evaluate the molecular mechanism of OS dedifferentiation.

OS exhibits genomic heterogeneity including diverse copy number gains and losses, due to its high chromosomal instability^34^. This complexity has always been a hurdle confounding our knowledge about OS biology. However, we utilized three OS cell line with various genetic status of p53 to construct a conceptual framework of OS dedifferentiation. It was well established that p53 could block the generation of human and mouse pluripotent cells from suboptimal parental cells, and the abrogation of p53 increased the efficiency of reprogramming^35,36^. These three OS cell lines presented different efficiency of dedifferentiation, calling our attention to the possible link between p53-mediated regulator and reprogramming.

**Fig. 7:**
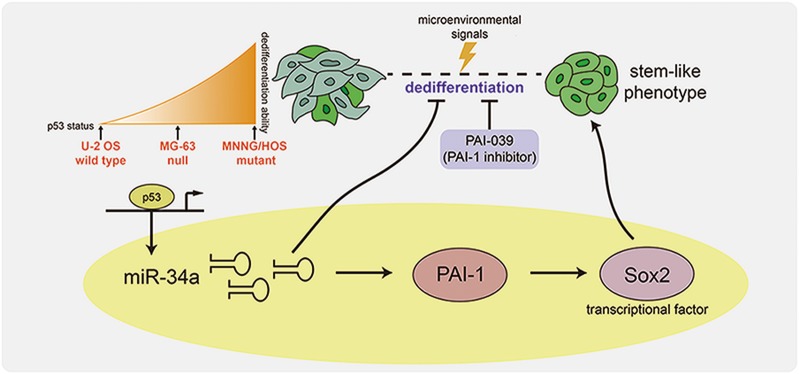
Schematic representation of miR-34a–PAI-1–Sox2 regulatory axis on the dedifferentiation of OS. During the TGFβ1-induced dedifferentiation, OS cells acquired stem-cell-like phenotypes, along with elevated expression level of Sox2 and miR-34a. miR-34a could downregulate PAI-1, which in turn suppressed Sox2 expression level to repress the dedifferentiation of OS

To our satisfaction, this study highlighted the contribution of p53-targeted miR-34a in OS cell reprogramming and completed the picture on how this miRNA regulating an exact crosstalk network during this process. miRNAs are a class of small, single-stranded RNA molecules ranging from 18 to 25 nucleotides in length. miRNA signatures are detected in diverse types of cancers such as sarcoma, breast and prostate cancer^37–39^. Compared to normal tissues, the expression level of miR-34 family was decreased in tumor samples and displayed minimal deletions and epigenetic inactivation^8^. Low miR-34a and miR-192 correlated with disappointing prognosis in OS patients^40^. During the dedifferentiation, miR-34a was confirmed to be activated and attempted to downregulate its downstream targets, PAI-1, to repress this process. These findings bring an insight for the development of diagnostic and prognostic markers, as well as targeted therapeutics for OS (Fig 7).

Emerging evidence demonstrated that miR-34a served as an inhibitory role and was a promising therapeutic target for OS. Pre-miR-34a prodrug could dramatically repress the proliferation of OS cells and was well tolerated in orthotopic xenograft mouse models^41^. A remarkable study demonstrated that combination treatment with miR-34a and doxorubicin presented more effective inhibition of cell proliferation in vitro and OS tumor growth in an orthotopic xenograft tumor mouse model compared to single drug treatment^42^. Of particular note, this treatment showed great synergistic effects without causing any toxicity to the liver and kidney of mice. The utility of miR-34a to combat malignant OS appears to have great potential and is necessary for further examination before it could be applied in human whole-body system.

A key novel significance of our work was first illustrating the precise function of PAI-1 linked with p53–miR-34a pathway in the regulation of OS dedifferentiation and provided some clinical possibilities. PAI-1 is the main inhibitor of tissue-type (t-PA) and urokinase-type (u-PA) plasminogen activator, involving in cell migration and tumor development^43^. In many respects, PAI-1 is required for promoting tumor growth through the inhibition of apoptosis^44,45^. PAI-1–deficient murine fibrosarcoma cells showed significantly suppressed tumorigenicity in nude mice^46^. As being highly expressed in several malignancies, PAI-1 is also associated with tumor invasion, metastasis and angiogenesis^47–49^. To date, the role of PAI-1 is yet limited understood in the regulation of cancer properties, especially in OS.

We thus far resolved a distinct pathway in the regulation of OS stemness by posing the correlation of PAI-1 and Sox2. Mounting evidence has established the essential role of Sox2 in the maintenance of TICs with stem cell properties in OS. Sox2-depleted OS cells reduced sphere formation and attenuated tumor formation in the xenograft assay. Overexpression of Sox2 enhanced osteosphere formation and increase adipogenic differentiation, while Sox2-depleted cells only underwent osteogenic differentiation^50,51^. A striking feature of our findings was that heterogeneous subtypes of OS cells were observed in BEM and stem-like OS cells could be identified with Sox2^+^ staining. The BEM-OS model rested in culture plate without flowing medium, and consequently, the oxygen and nutrients were unevenly distributed. We supposed that OS cells under unsatisfactory microenvironment were likely to acquire a poorly differentiated phenotype for survival and better adaptation. Our previous study indicated the role of hypoxia in regulating OS dedifferentiation^4^. Other research also showed that oxygen or glucose shortage contributed to the enrichment of CSCs, and their flexible modulation between different metabolic profiles^52^. To identify the relationship between PAI-1 expression and differentiation degree, we examined the mRNA expression level of PAI-1 under hypoxic environment. Result demonstrated that PAI-1 was significantly elevated, consistent with the upregulation during OS dedifferentiation (Supplementary Fig. S7). Besides, PAI-1^+^/Sox2^+^ cells could be detected around the necrotic area in the histological section of BEM (Fig. 6c). Compared to the well differentiated ones, this poorly differentiated subpopulation with active PAI-1 expression level might undergo reprogramming process, and result in the remodeling of cellular morphology and the stimulation of stemness-related gene, Sox2.

As an indole oxoacetic acid PAI-1 inhibitor, PAI-039 activity was extensively and successfully tested in pre-clinical rat and canine models of acute arterial thrombosis^43^. The inhibition of PAI-1 by PAI-039 or siRNA mediated gene silencing could reduce the self-renewal capability and increase the radiosensitivity of head and neck TICs^26^. In cancer therapy, PAI-039 had been administered by oral gavage to athymic mice bearing human bladder cancer cell line T24 xenografts and human cervical cancer HeLa cell xenografts. This treatment caused a reduction in tumor angiogenesis and cell proliferation, and an induction in apoptosis, leading to a marked decrease in tumor volume^53^. The notion that PAI-1 as a potential therapeutic target is further strengthened by the work shown in this study. Doxorubicin treatment combined with PAI-039 exhibited significant synergy in the suppression of OS cell proliferation (Supplementary Fig. S8a, b). We could speculate a possible targeted treatment strategy, such that the combination of lower-dose chemotherapy drugs to kill cancer cells with PAI-1 inhibitor to attenuate the CSC subpopulation in OS.

Uncovering the relationship between specific signaling pathways and microenvironmental signals in dedifferentiation will shed light on the solutions for tumor eradication. We laid a solid foundation that miR-34a–PAI-1–Sox2 axis could be a pivotal target involved in OS dedifferentiation. What seemed to be noteworthy is the underlying p53–miR-34a interaction in controlling OS cell reprogramming, of which we should continue to explore.

## Electronic supplementary material

Supplementary Table

Supplemental Figure S1

Supplemental Figure S2

Supplemental Figure S3

Supplemental Figure S4

Supplemental Figure S5

Supplemental Figure S6

Supplemental Figure S7

Supplemental Figure S8

Supplementary figure legends
