# Supplementary material for: miR-34a exerts as a key regulator in the dedifferentiation of osteosarcoma via PAI-1–Sox2 axis
Source: Cell Death Dis. 2018 Jul 10;9(7):777. doi: 10.1038/s41419-018-0778-4 (PMC6039486; doi:10.1038/s41419-018-0778-4)
Supplement: Supplementary file 1 — Supplementary Table [file 41419_2018_778_MOESM1_ESM.docx]

**Supplementary**

**Table S1. The oligonucleotides used in this work.**

| Name | Sequence (5’-3’) |
| --- | --- |
| hsa-miR-34a-5p mimic double strand | UGGCAGUGUCUUAGCUGGUUGU |
|  | ACAACCAGCUAAGACACUGCCA |
| hsa-miR-34a-5p inhibitor single strand | ACAACCAGCUAAGACACUGCCA |
| siPAI-1 1 | TGACCGACATGTTCAGACA |
| siPAI-1 2 | GAGCCAGATTCATCATCAA |
| siPAI-1 3 | CTGACAACAGGAGGAGAAA |

**Table S2. Primer sequences for qRT-PCR.**

| Name | Sequence (5’-3’) |
| --- | --- |
| miR-34a-5p RT primer | CTCAACTGGTGTCGTGGAGTCGGCAATTCAGTTGAGACAACCAG |
| miR-34a-5p forward primer | TCGCCTGGCAGTGTCTTAGCT |
| Reverse primer | CTCAACTGGTGTCGTGGAGTCGGC |
| U6 forward primer | CGCTTCGGCAGCACATATAC |
| U6 RT primer | TTCACGAATTTGCGTGTCAT |
| pri-miR-34a-F | CCTCCAAGCCAGCTCAGTTG |
| pri-miR-34a-R | TGACTTTGGTCCAATTCCTGTTG |
| pri-miR-34b/c-F | GCTCTTTGTCCCTCCTGCTAGA |
| pri-miR-34b/c-R | GTGGGCGGTCCCTGAAG |
| GAPDH-F | GGAGCGAGATCCCTCCAAAAT |
| GAPDH-R | GGCTGTTGTCATACTTCTCATGG |
| PAI-1-F | GCATGTTCATTGCTGCCCCTT |
| PAI-1-R | CCAGGGAGAACTTGGGCAGAA |
| SOX2-F | AGCTACAGCATGATGCAGGA |
| SOX2-R | GGTCATGGAGTTGTACTGCA |
| β-actin-F | ACTGGAACGGTGAAGGTGAC |
| β-actin-R | AGAGAAGTGGGGTGGCTTTT |
| PPARγ-F | ATGGAGTCCACGAGATCATT |
| PPARγ-R | CGCAGGCTCTTTAGAAACTC |
| aP2-F | AACCTTAGATGGGGGTGTCCTG |
| aP2-R | TCGTGGAAGTGACGCCTTTC |
| GLUT4-F | AGGATCGGTTCTTTCATCTTCGC |
| GLUT4-R | GTTCCCCATCTTCGGAGCCTA |
| LPL-F | AGTGGCCAAATAGCACATCC |
| LPL-R | CCGAAAGATCCAGAATTCCA |
| JAG1-F | CTGGCGGCTGGGAAGGAACAAC |
| JAG1-R | GCAGTCGGGCCCAGCAAAACC |
| NOTCH1-F | GCAGAGGCGTGGCAGACTAT |
| NOTCH1-R | TGGCACGATTTCCCTGACCA |
| HES1-F | GTCAACACGACACCGGATAAACCA |
| HES1-R | TGGAATGCCGCGAGCTATCTTTCT |
| HEY1-F | CGAGGTGGAGAAGGAGAGTG |
| HEY1-R | CTGGGTACCAGCCTTCTCAG |
| SNAI1-F | TGCGGGCCCACCTCCAGAC |
| SNAI1-R | CCCCCGACAAGTGACAGCCATTAC |
| SNAI2-F | ACGCCTCCAAAAAGCCAAACTACA |
| SNAI2-R | CTTCAGGGCGCCCAGGCTCACATA |
| PDGFRB-F | GACACCAGCTCCGTCCTCTA |
| PDGFRB-R | GGCTGTCACAGGAGATGGTT |
