# Supplementary figures and images for: miR-34a exerts as a key regulator in the dedifferentiation of osteosarcoma via PAI-1–Sox2 axis
Source: Cell Death Dis. 2018 Jul 10;9(7):777. doi: 10.1038/s41419-018-0778-4 (PMC6039486; doi:10.1038/s41419-018-0778-4)

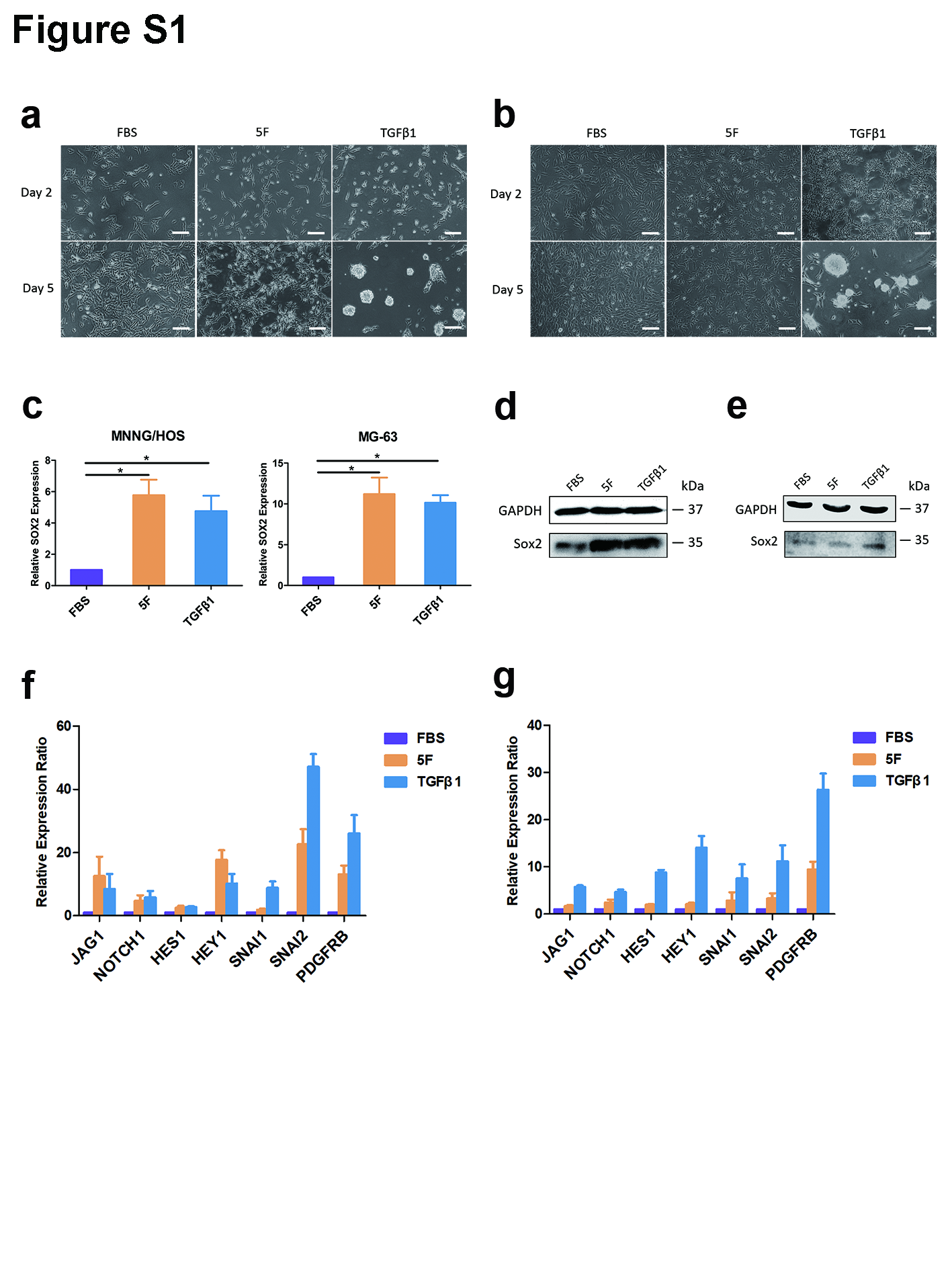

Supplement: Supplementary file 2 — Supplemental Figure S1 [file 41419_2018_778_MOESM2_ESM.tif]

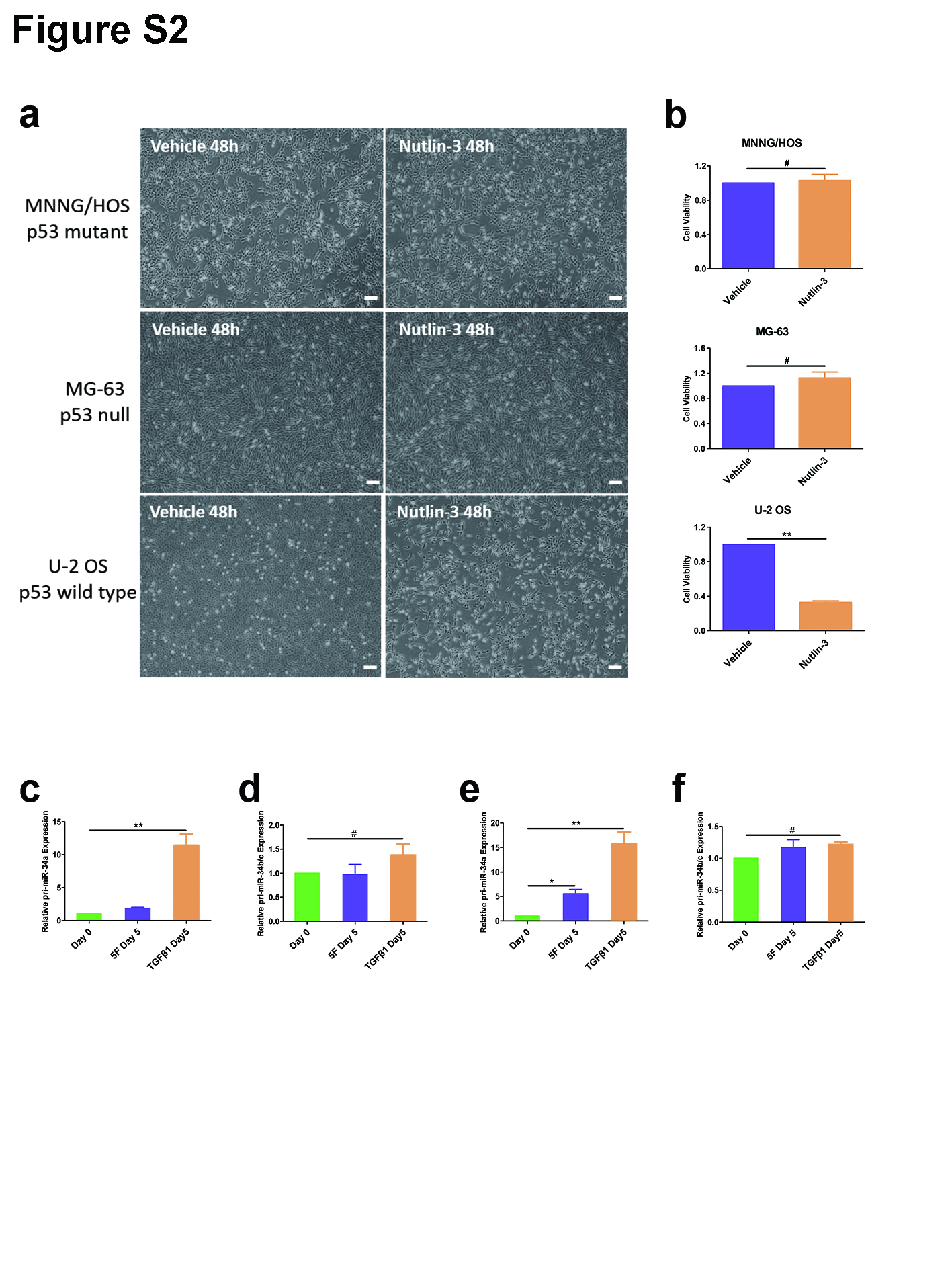

Supplement: Supplementary file 3 — Supplemental Figure S2 [file 41419_2018_778_MOESM3_ESM.tif]

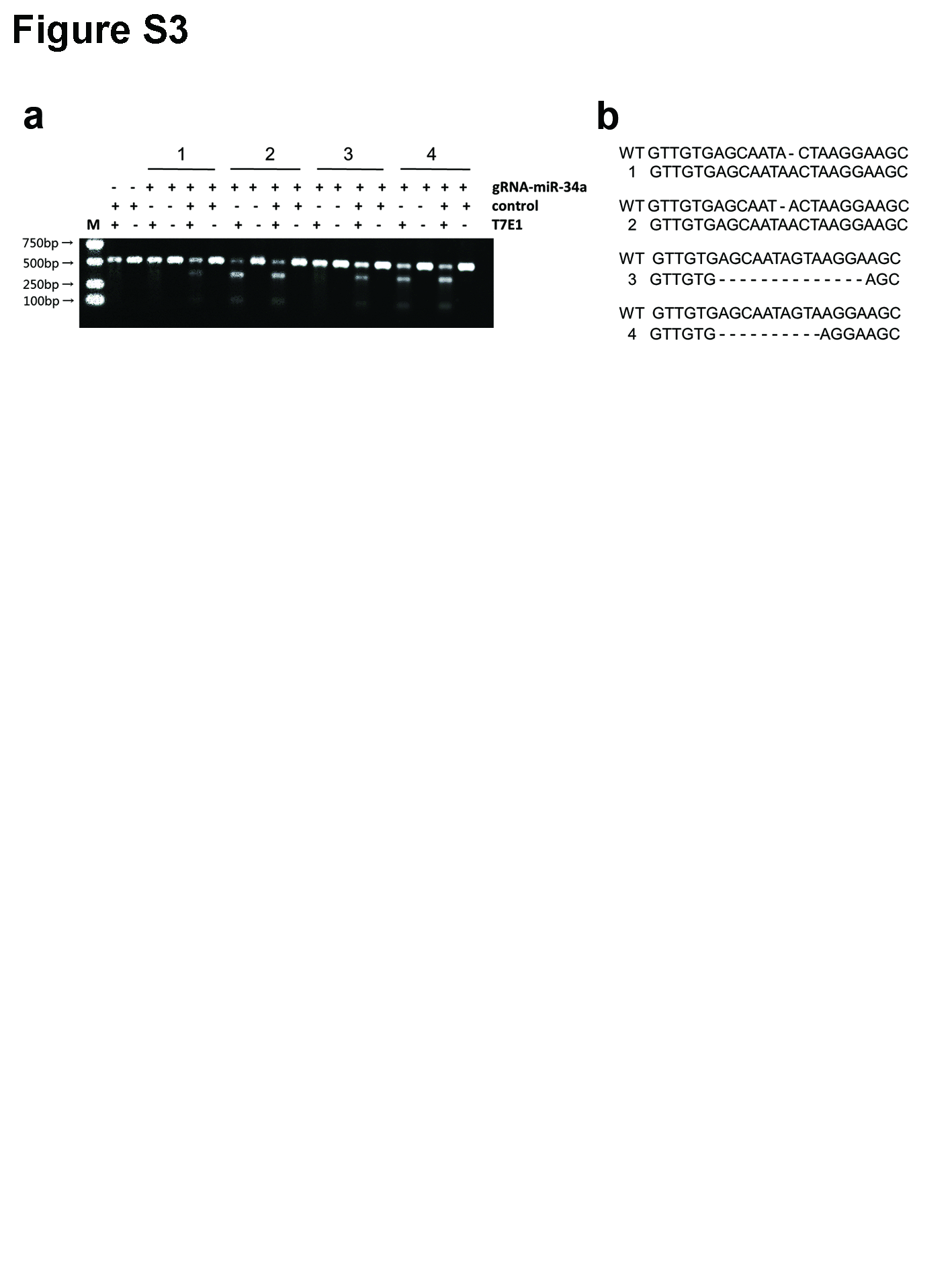

Supplement: Supplementary file 4 — Supplemental Figure S3 [file 41419_2018_778_MOESM4_ESM.tif]

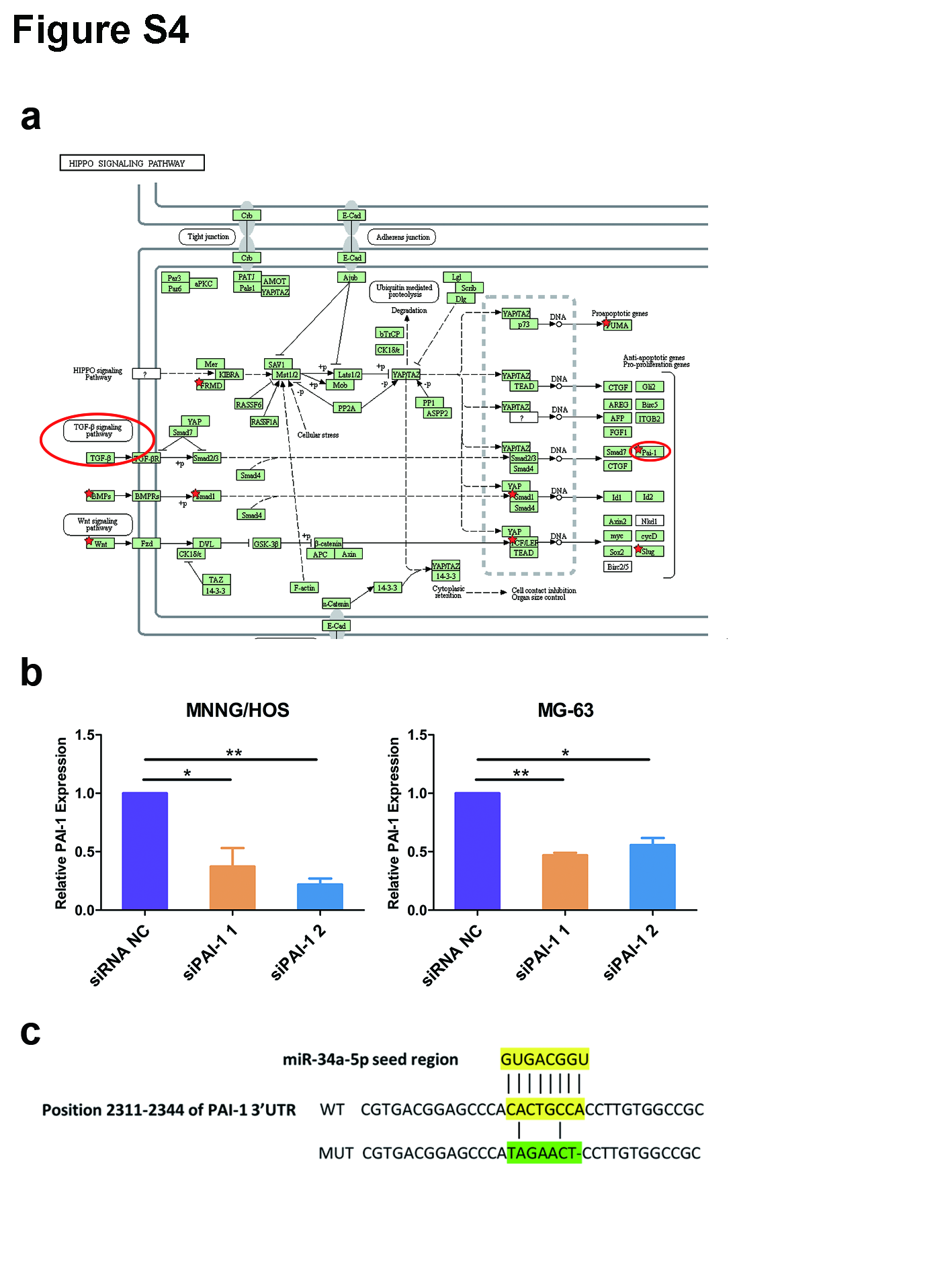

Supplement: Supplementary file 5 — Supplemental Figure S4 [file 41419_2018_778_MOESM5_ESM.tif]

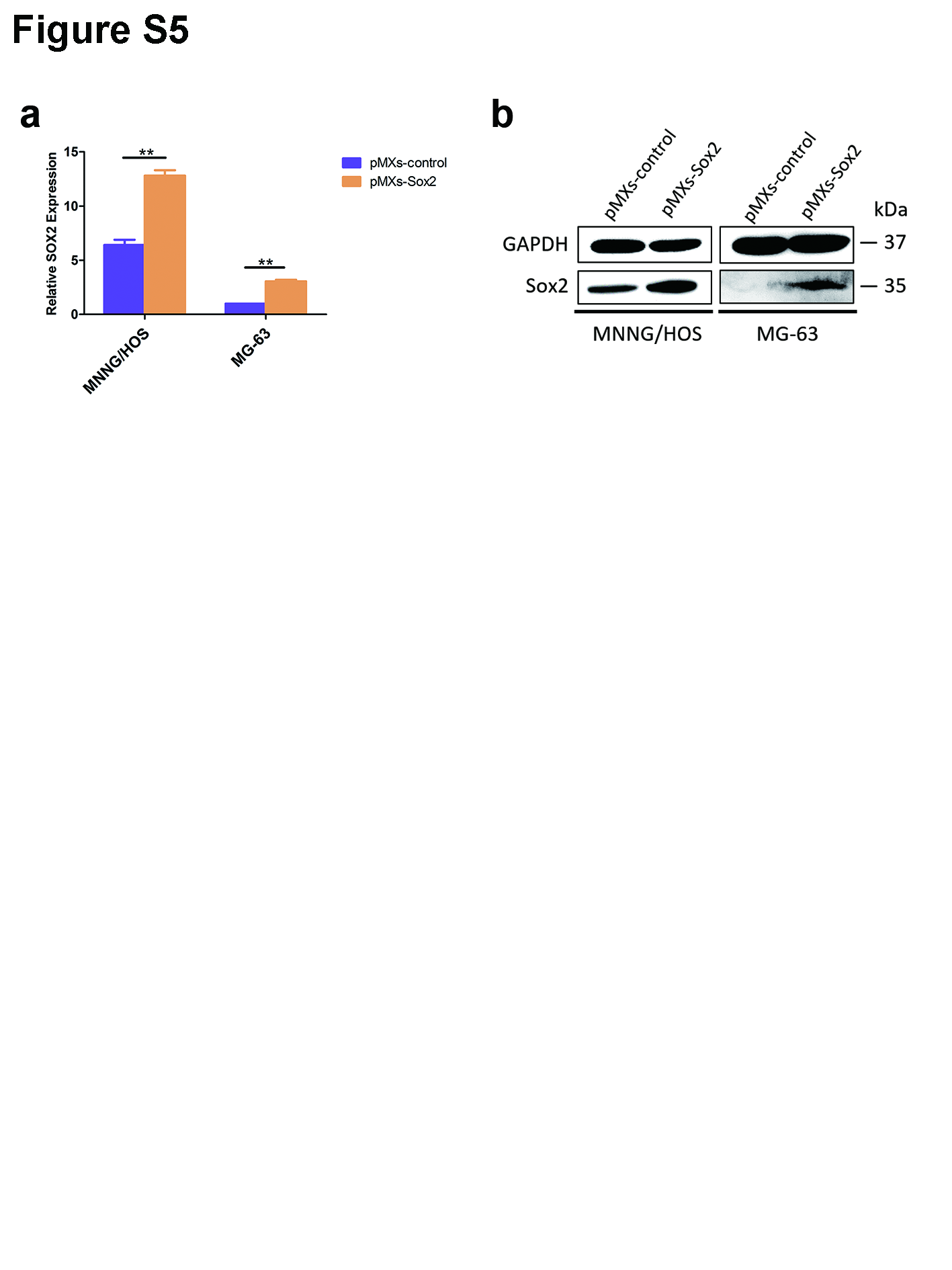

Supplement: Supplementary file 6 — Supplemental Figure S5 [file 41419_2018_778_MOESM6_ESM.tif]

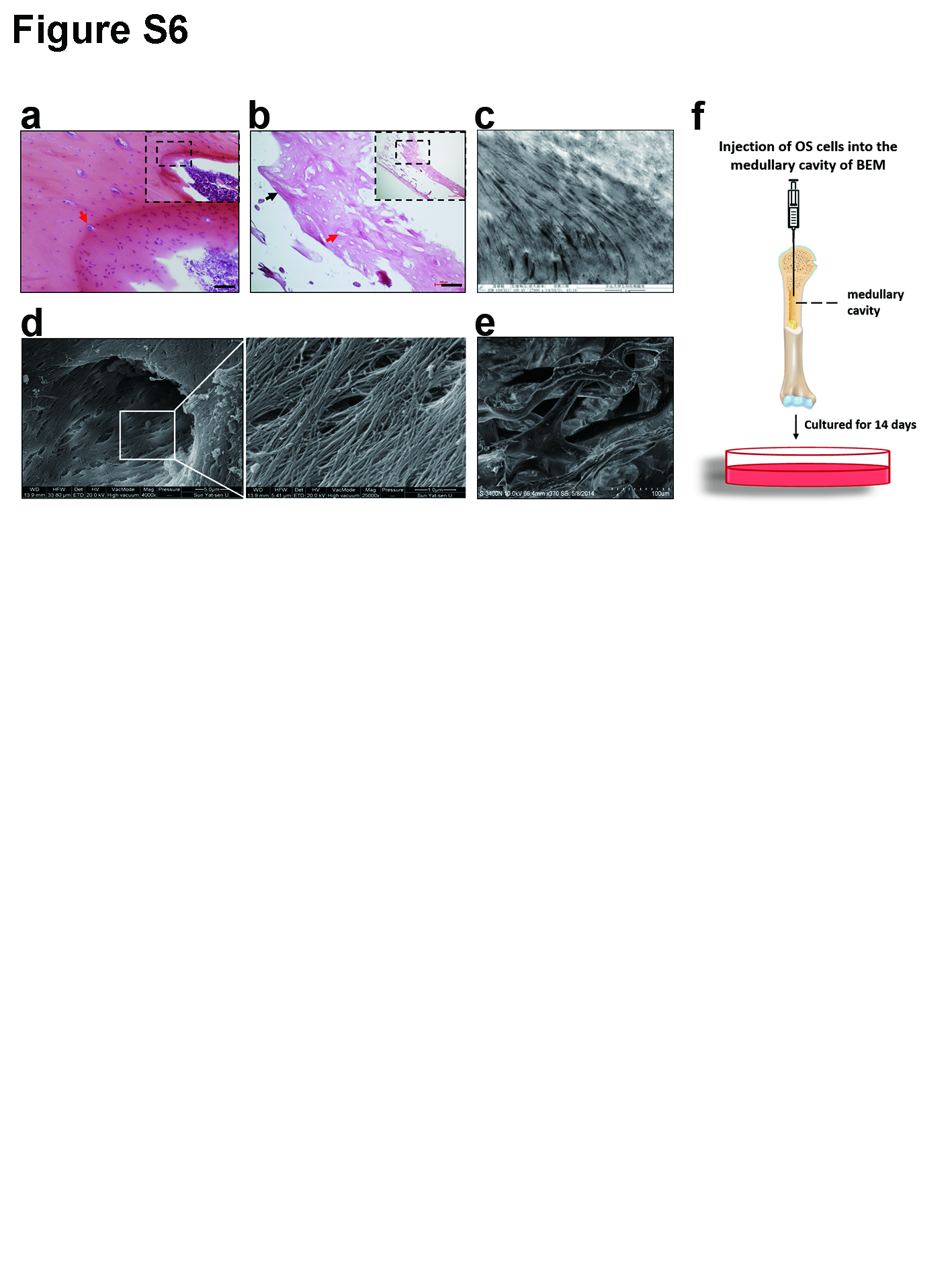

Supplement: Supplementary file 7 — Supplemental Figure S6 [file 41419_2018_778_MOESM7_ESM.tif]

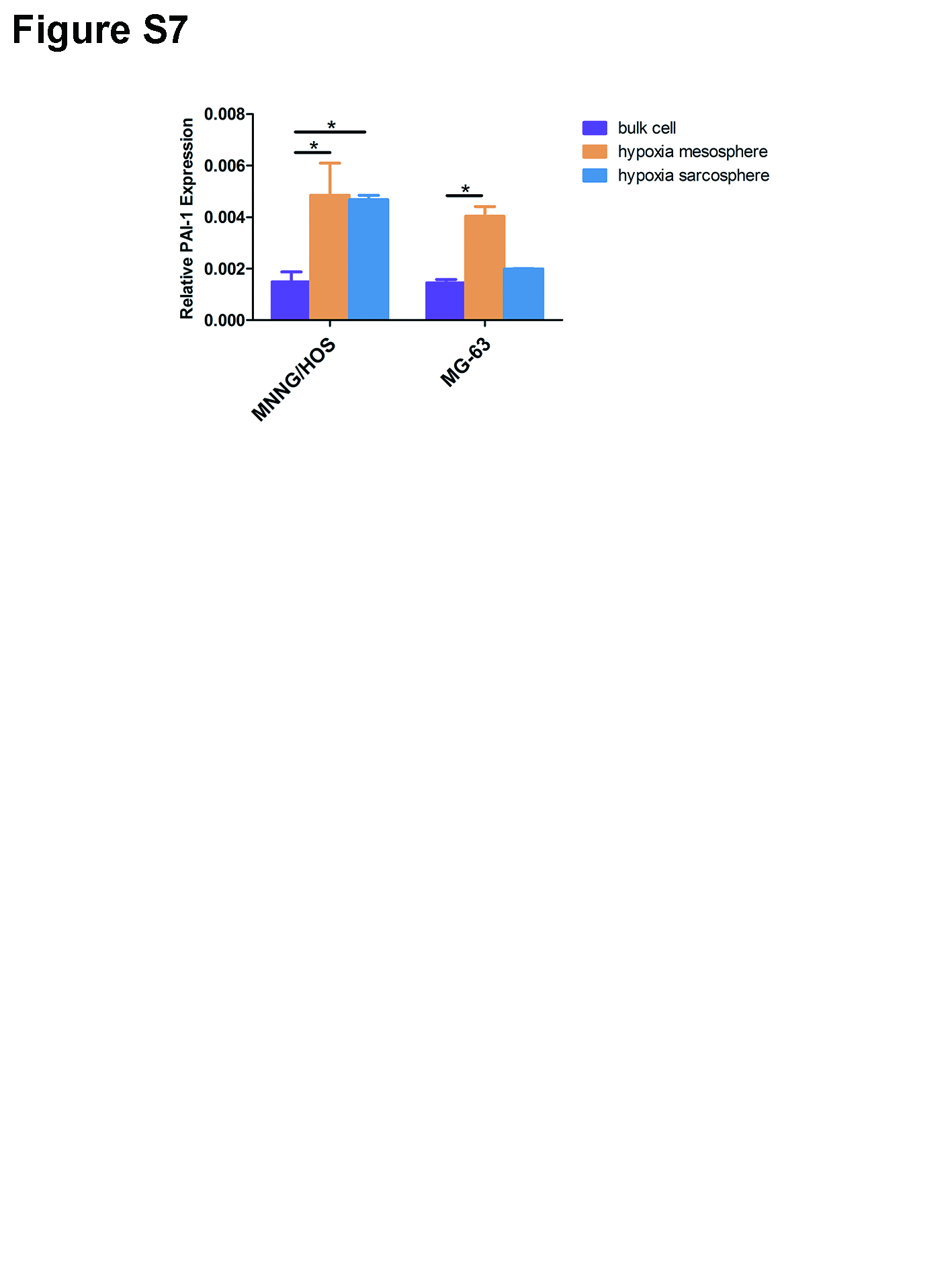

Supplement: Supplementary file 8 — Supplemental Figure S7 [file 41419_2018_778_MOESM8_ESM.tif]

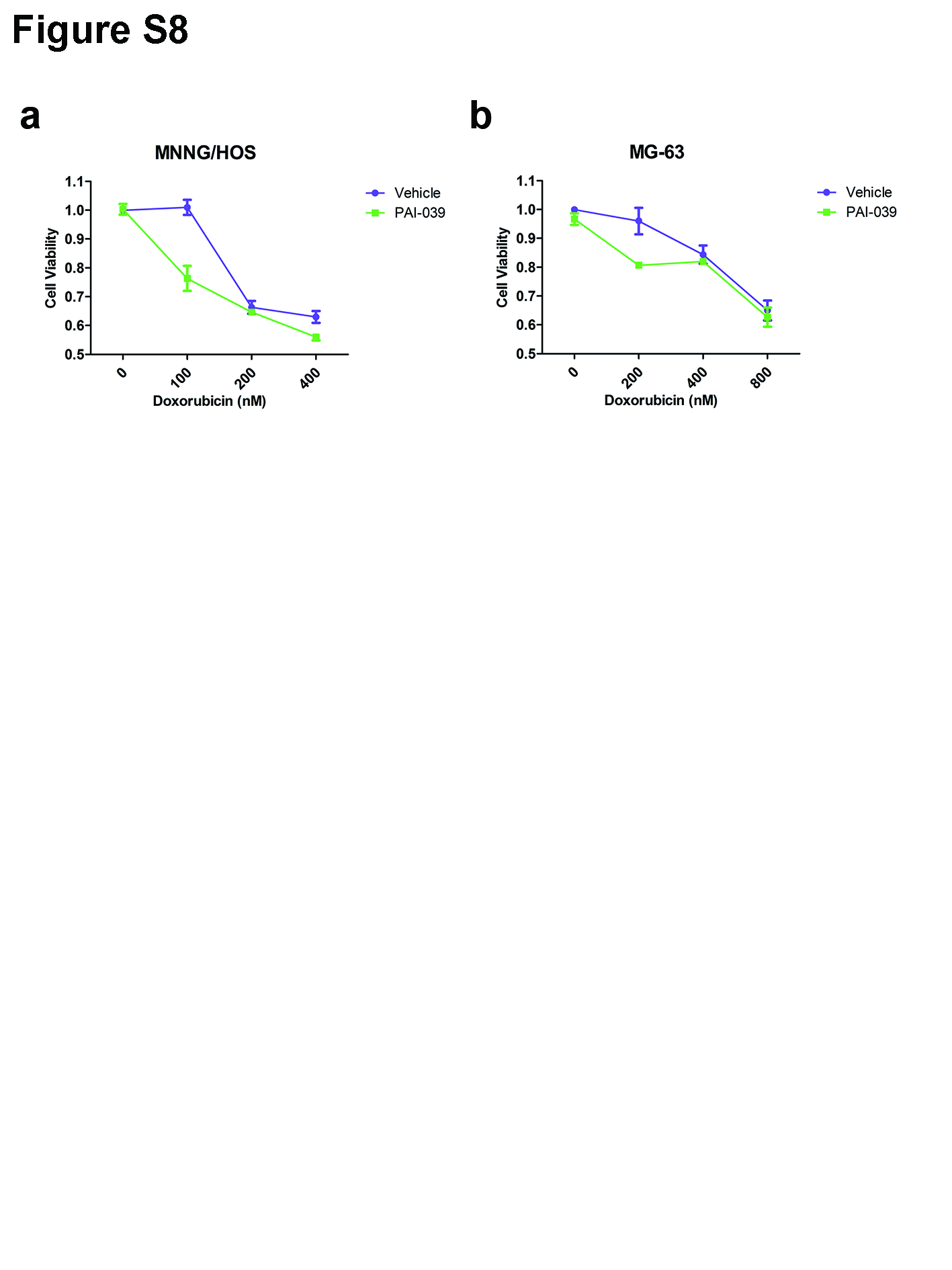

Supplement: Supplementary file 9 — Supplemental Figure S8 [file 41419_2018_778_MOESM9_ESM.tif]
