# Supplementary material for: miR-34a exerts as a key regulator in the dedifferentiation of osteosarcoma via PAI-1–Sox2 axis
Source: Cell Death Dis. 2018 Jul 10;9(7):777. doi: 10.1038/s41419-018-0778-4 (PMC6039486; doi:10.1038/s41419-018-0778-4)
Supplement: Supplementary file 10 — Supplementary figure legends [file 41419_2018_778_MOESM10_ESM.docx]

**Supplementary Figure Legends**

**Figure S1. Morphology and gene expression of OS cells in different culture conditions.**

(a, b) MNNG/HOS (a) and MG-63 (b) cells cultured in DF containing 5% FBS or serum-free medium with or without TGFβ1. Scale bar = 100 μm. (c-e) qRT-PCR (c) and western blot (d, e) analysis of stem-cell markers Sox2 expression during the dedifferentiation of OS cells. (f, g) qRT-PCR analysis of upregulated genes in the process of MNNG/HOS (f) and MG-63 (g) dedifferentiation. *P<0.05.

**Figure S2. Nutlin-3 treatment could induce a growth-inhibiting state in U-2 OS cell.**

(a, b) Phase contrast images (a) and quantitative results (b) for the proliferation of each cell line after Nutlin-3 treatment were shown as cell numbers. Scale bar = 100 μm. (c-f) qRT-PCR analysis of pri-miR-34a (c, e) and pri-miR-34b/c (d, f) expression levels in MNNG/HOS (c, d) and MG-63 (e, f) cells in the process of dedifferentiation. **P < 0.01, #P＞0.05.

**Figure S3. miR-34a was successfully knockdowned via lenti-CRISPR system.**

(a) The T7EI assay was used to detect CRISPR/Cas-induced indels in U-2 OS cells. (b) DNA sequences of the targeting sites in the miR-34a-depleted U-2 OS cells are represented.

**Figure S4. PAI-1 was a potential target of miR-34a.**

(a) KEGG pathway map of differentially expressed genes participating in the dedifferentiation of OS. (b) Inhibition of PAI-1 in MNNG/HOS and MG-63 cells. *P<0.05, **P<0.01. (c) Schematic showing putative miR-34a target site in the 3’UTR of PAI-1. Top, predicted miR-34a-5p regulatory elements (seed sequences in upper case) in the PAI-1 3’UTR; bottom, sequences of wild-type (WT) or mutant (MUT) PAI-1 3’UTR luciferase reporters.

**Figure S5. Overexpression of Sox2 in MNNG/HOS and MG-63 cells.**

(a, b) Sox2 expression levels were verified by qRT-PCR (a) and western blot (b) after transfected with pMXs-Sox2 overexpressing construct. **P<0.01.

**Figure S6. Establishment of three-dimensional bone extracellular matrix model.**

(a) H&E staining of the native bone from mouse tibia and decellularized bone from mouse tibia. Black arrow indicated that collagen in the bone plate is arranged in parallel. Red arrows proved that no bone cells were presented in the bone lacunae between the bone plates in the decellularized BEM. Scale bar = 100 μm. (b) Transmission electron microscopy analysis of the decellularized mouse tibia. (c) Scanning electron microscopy analysis of the native mouse tibia and decellularized mouse tibia. (d) Experimental schema for the BEM-OS models.

**Figure S7.** **Upregulation of PAI-1 might be responsible for OS dedifferentiation induced by hypoxia.**

qRT-PCR analysis of PAI-1 expression level in MNNG/HOS and MG-63 cells during hypoxia-induced dedifferentiation. *P<0.05.

**Figure S8. Effect of combination treatment of doxorubicin and PAI-039 on OS cell viability.**

(a, b) MNNG/HOS (a) and MG-63 (b) cells were treated with various concentrations of doxorubicin for 48 h.
